# Establishment of Nucleic Acid Amplification Technology for the Detection of *Mycoplasma* in Biological Products

**DOI:** 10.3390/molecules31111794

**Published:** 2026-05-23

**Authors:** Ying Guo, Xi Qin, Jing Zhang, Hua Bi, Shuting Hou, Youxue Ding, Dening Pei, Xiang Li, Yue Pan, Xiaoliang Sun, Chenggang Liang

**Affiliations:** 1 National Institute for Food and Drug Control, No. 31 Huatuo St., Daxing District, Beijing 100050, China; guoying@nifdc.org.cn (Y.G.); qinxi@nifdc.org.cn (X.Q.); bihua@nifdc.org.cn (H.B.); xiaohouxj@sina.com (S.H.); dingyouxue@nifdc.org.cn (Y.D.); lix@nifdc.org.cn (X.L.); 2 Yeasen Biotechnology (Shanghai) Co., Ltd., Building 1, Lane 166, Tianxiong Road, International Medical Park, Pudong New Area, Shanghai 201318, China; zhangjing@yeasen.com; 3 College of Science, Xi’an Jiaotong-Liverpool University, Room 111, Ren’ai Road, Dushu Lake Science and Education Innovation District, Suzhou Industrial Park, Suzhou 215123, China; 18728300996@163.com

**Keywords:** *Mycoplasma* contamination, detection, validation, sensitivity, biological products

## Abstract

Currently, the most commonly used methods for detecting *Mycoplasma* are the culture method and the indicator cell culture method. However, both approaches exhibit low sensitivity and are incapable of detecting low-concentration contamination. In addition, the detection period may extend up to 28 days, which is unsuitable for rapid screening and may delay timely contamination control measures. To address these limitations, a *Mycoplasma* detection method based on nucleic acid amplification technology (NAT) was developed following a comparative analysis of gene sequences from various *Mycoplasma* species. The method was validated with respect to its detection performance and its applicability to biological product samples. DNA was extracted from *Mycoplasma*-contaminated samples using a magnetic bead-based nucleic acid extraction method. Universal primers were designed based on the highly conserved *16S rRNA* gene sequence of *Mycoplasma*, and amplification was performed using multiplex quantitative PCR (qPCR) with fluorescent probes. The limit of detection (LOD) was established based on statistics of 24 replicates. Method specificity and robustness were evaluated according to the guidelines set by the International Council for Harmonisation of Technical Requirements for Pharmaceuticals for Human Use (ICH Q2), while sample applicability was assessed in accordance with the European Pharmacopoeia (EP) <2.6.7>. The NAT-based *Mycoplasma* detection method enabled rapid, qualitative identification of *Mycoplasma* contamination. The validated LOD was 10 CFU/mL, and the method met predefined requirements for sensitivity, specificity, and robustness. To assess applicability, real biological product samples, including monoclonal antibodies, antibody fusion proteins, bispecific antibodies, and trispecific antibodies, were spiked with 10 CFU/mL of standard *Mycoplasma* strains. All spiked samples tested positive. These findings confirm that the NAT-based *Mycoplasma* detection method is suitable for process control and product release testing in the production of biological products.

## 1. Introduction

In recent years, driven by growing market demand and rising industry standards, the biological products industry has experienced rapid development. Concurrently, the production, quality control, and safety of biological products have become focal points of regulation and supervision by governments worldwide. During the manufacturing process of biological products, such as therapeutic monoclonal antibodies, recombinant proteins, and cell therapy products, *Mycoplasma* can readily enter the cell culture system through raw materials (e.g., serum, cell banks), production equipment, personnel, or environmental exposure. *Mycoplasma* is a common contaminant in biological products and is difficult to eliminate. Its presence may reduce the potency and stability of products, and once contamination is detected, entire production batches may need to be discarded. Currently, several methods are available for *Mycoplasma* detection, including culture-based methods, indicator cell culture methods (e.g., DNA staining techniques), nucleic acid amplification techniques (NAT), enzyme-linked immunosorbent assays (ELISA), and biochemical assays [1]. Among these, the culture method and the indicator cell culture method are widely accepted by pharmacopeias in many countries for detecting *Mycoplasma* contamination in biological products. However, due to their low sensitivity, these methods are inadequate for detecting low-level contamination. Additionally, their long detection times, which can extend up to 28 days, do not meet the requirements for rapid screening and may delay contamination management [2]. Furthermore, because cell therapy products often require fresh infusion or immediate freezing, the lengthy turnaround of culture-based methods is incompatible with the time-sensitive nature of these therapies. As a result, there is an urgent need for rapid *Mycoplasma* detection methods that support timely product release, particularly in the field of cell and gene therapy [3].

In addition to the traditional culture method and indicator cell methods [4], the United States Pharmacopeia (USP) <63>, European Pharmacopoeia (EP) <2.6.7>, and Japanese Pharmacopoeia (JP) now recommend nucleic acid amplification technology (NAT) as an alternative for *Mycoplasma* detection [5,6,7]. When sufficiently validated and shown to be comparable to traditional methods in sensitivity, NAT can replace the culture method for both in-process control and release testing.

Polymerase chain reaction (PCR) is a widely used and well-established nucleic acid amplification technique which is applicable to rapid *Mycoplasma* detection. The design strategy for mycoplasma PCR primers and probes has evolved from an initial emphasis on broad applicability to a current focus on high coverage, specificity, and sensitivity. This shift has been driven by evolving detection requirements and continuous advancements in pharmaceutical quality control. Early primer designs targeted highly conserved regions of the *16S rRNA*. The incorporation of degenerate primers helped accommodate interspecies variations, enabling broad coverage across the *Mollicutes*. However, these approaches often yielded long amplification products along with poor sensitivity and high non-specific background noise [8]. With progress in bioinformatics, researchers optimized primer coverage and specificity by targeting *Mycoplasma*-specific conserved regions and improved detection specificity through fluorescence-based methods and probe chemistry. Still, achieving a balance between broad species coverage and high sensitivity remained challenging [9]. Research efforts subsequently branched into two main directions. One direction aligns with pharmacopeial standards by designing strain-specific primers and probes. This focused strategy enables excellent specificity and sensitivity due to significant sequence divergence among *Mycoplasma* species [10]. The other direction utilizes multiplex detection with three or more primer-probe sets to broaden species coverage while maintaining specificity, though often at the cost of reduced sensitivity [11]. This study presents a three primer-probe set that achieves coverage of 183 species within *Mollicutes* while maintaining high specificity. Validation using pharmacopoeial strains confirmed single-copy detection sensitivity, supporting its use as a precise and efficient tool for quality control and regulatory applications (Table 1).

Moreover, the European and Japanese Pharmacopoeias provide detailed and largely consistent guidelines for validating *Mycoplasma* nucleic acid amplification testing (NAT). In this study, a NAT-based method was established for process control and rapid release testing of *Mycoplasma* contamination in biological products. The method was evaluated for limit of detection (LOD), specificity, and robustness, and its applicability was confirmed in various real biological product samples, in alignment with relevant national and international regulatory requirements (Table 2).

## 2. Results

### 2.1. Species Coverage Analysis of the Mycoplasma NAT Method (Fluorescence Probe qPCR Method)

**Figure 1:**
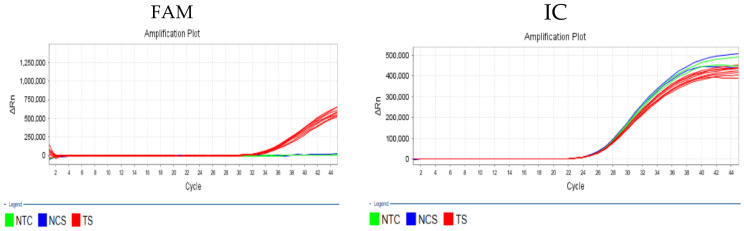
Detection results of *Mycoplasma orale* at 10 CFU/mL.

To evaluate the species coverage of the *Mycoplasma* NAT method using fluorescence probe-based qPCR, the primer sequences provided by the manufacturer were compared against genomic sequences from public databases. In this method, the primer and probe sequences are aligned with the *16S rRNA* genome database. A *Mollicutes* species is considered specifically detectable if there is no more than one base mismatch at the 3 ‘end of the primer and no more than one mismatch in the probe sequence. Based on sequence alignment results, this method can detect at least 183 species of *Mollicutes*, including 120 species of *Mycoplasma*, 9 species of *Ureaplasma*, 33 species of *Spiroplasma*, and 7 species of *Acholeplasma* (typical graphics see Figure 1).

### 2.2. Limit of Detection (LOD)

According to the instructions provided with the standard strains, the *Mycoplasma* standard dry powder was reconstituted using 1× PBS buffer. Nucleic acids were extracted from the resulting *Mycoplasma* suspension following the procedures described in Section 4.3. “Sample Nucleic Acid Extraction” and Section 4.4. “qPCR Detection”. The experiment was conducted over three consecutive days, with eight independent nucleic acid extractions performed each day. Each extraction was followed by one qPCR assay, resulting in a total of 24 test data points. NCS and NTC samples were included in each experiment. All NCS and NTC results were within acceptable limits, and all 24 *Mycoplasma* test results were positive. This met the criterion that at least 23 out of 24 replicates must yield a positive result to confirm the method’s LOD performance (Table 3).

### 2.3. Specificity

#### 2.3.1. Sample Matrix Interference

Following the procedures in Section 4.3. “Sample Nucleic acid Extraction” and Section 4.4. “qPCR Detection,” nine commonly used sample matrices were tested: Dulbecco’s MEM, Ham’s F-10 Medium, M-199 Medium, Ham’s F-12K (Kaighn’s), RMIP-1640 Medium, McCoy’s 5A Medium, Ham’s F-12K supplemented with 1.0 mM/L Glutamine, L-15 Leibovitz Medium with 2.05 mM L-Glutamine, DMEM- low glucose (standard type), and the DNA diluent included in the kit. Both unspiked and spiked samples (10 CFU/mL *Spiroplasma citri*) were tested, along with PCS, NCS, and NTC samples in each experimental run. The PCS, NCS, and NTC results were all within acceptable ranges. The detection results of the unspiked matrices and diluents were negative, showing no cross-reactivity. All spiked samples yielded positive results, confirming that the method was not affected by matrix interference (Table 4).

#### 2.3.2. Cross-Reactivity

To evaluate specificity, 14 non-*Mollicutes* microbial strains were tested. These included *Clostridium perfringens*, *Clostridium acetobutylicum*, *Lactobacillus acidophilus*, *Streptococcus mutans*, *Streptococcus pneumoniae*, *Staphylococcus epidermidis*, *Acinetobacter baumannii*, *Enterobacter aerogenes*, *Micrococcus luteus*, *Pseudomonas aeruginosa*, *Candida albicans*, *Salmonella enterica* subspecies, *Bacillus subtilis*, and *Bacillus cereus*. Six engineered cell lines (HEK293, 293T, Vero, CHO, *E. coli*, and Sf9) were also included. All samples were tested using the same procedures outlined in Section 4.3 and Section 4.4. The PCS, NCS, and NTC samples were detected simultaneously in each experiment. The results indicated that the PCS, NCS, and NTC all performed within the acceptable range. All test samples showed negative results, indicating no cross-reactivity with the assay (Table 5).

### 2.4. Tolerance

#### 2.4.1. Freeze–Thaw Stability

**Figure 2:**
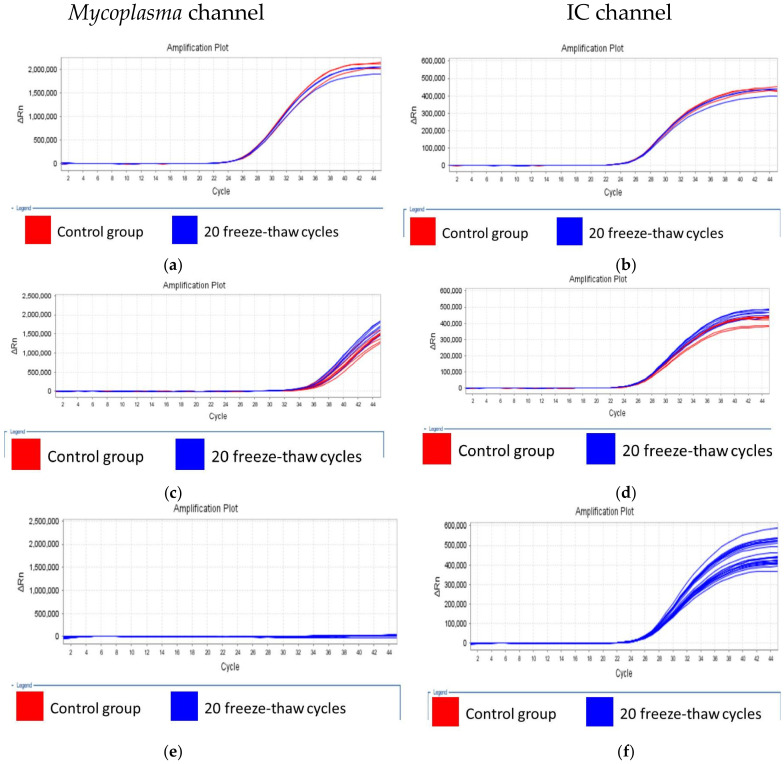
Results of freeze–thaw stability testing. The amplification curves of *Mycoplasma 16s rRNA* gene (**left column**) and internal reference gene (**right column**) from samples subjected to 20 freeze–thaw cycles and non-freeze–thaw controls were analyzed. The samples tested were as follows: 1000 copies/μ L positive control (PCS) (**a**,**b**), 1 copy/μ L PCS (**c**,**d**), and template free control (**e**,**f**).

All reagents used in the *Mycoplasma* NAT (fluorescence probe qPCR method) were subjected to freeze–thaw stability testing at −25 °C to −15 °C. For the positive control samples (PCSs) at a concentration of 1000 copies/μL, triplicate reactions were performed. At a concentration of 1 copy/μL, eight replicates were tested. A 24-well replicate test was conducted for the non-template control (NTC). After 0 and 20 freeze–thaw cycles, the NTC results remained within acceptable limits. The Ct values and fluorescence intensities of the positive templates at both concentrations showed no significant variation, indicating that the reagents maintained stability and performance after 20 freeze–thaw cycles (Table 6 and Figure 2).

#### 2.4.2. Thermal Accelerated Stability

**Figure 3:**
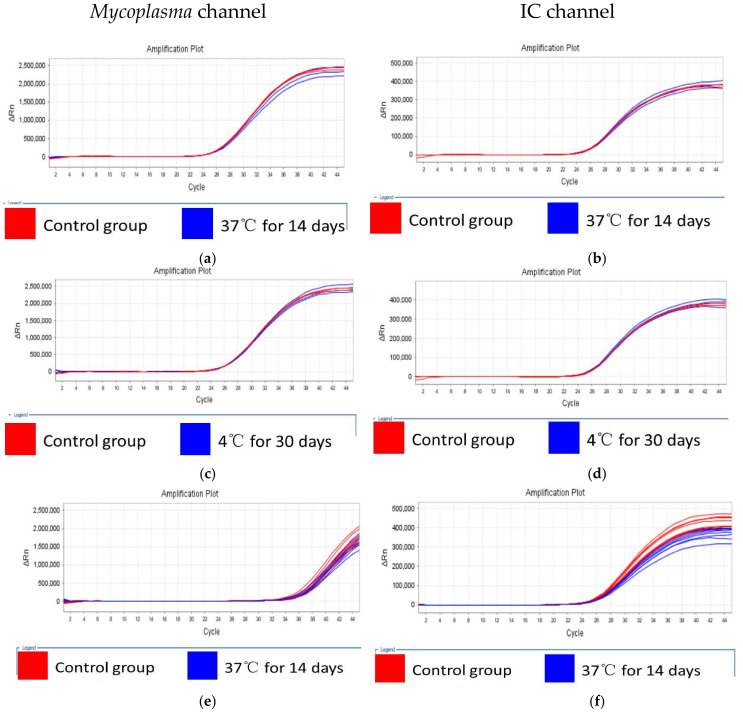

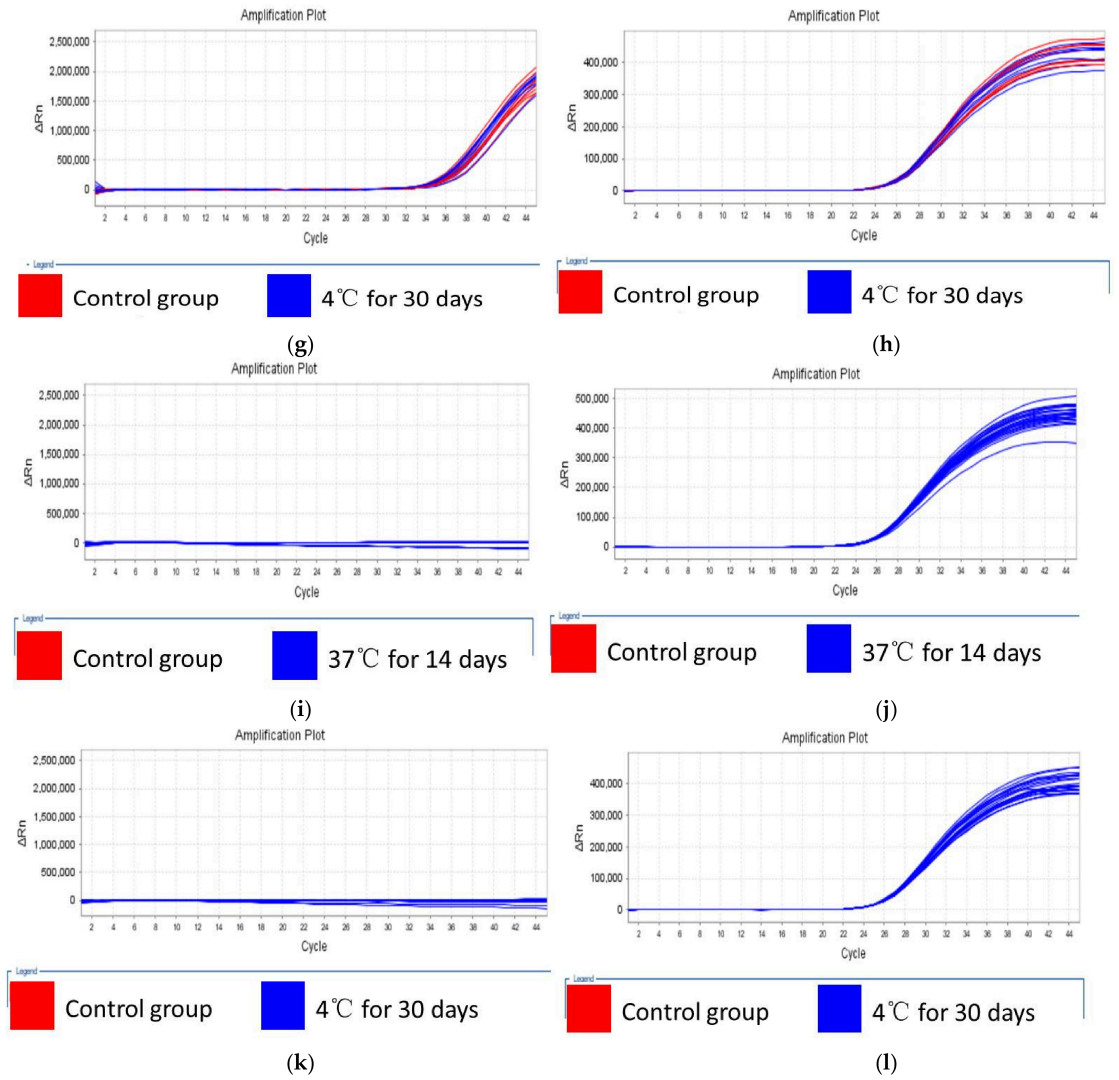
Thermal acceleration stability test results. The amplification curves of *Mycoplasma 16s rRNA* gene (**left column**) and internal reference gene (**right column**) of samples subjected to heat accelerated treatment at 37 °C for 14 days or 4 °C for 30 days, as well as untreated control samples, were as follows: 1000 copies/μ L positive control (PCS) (**a**–**d**), 1 copy/μ L PCS (**e**–**h**), and template free control (**i**–**l**).

All reagents were subjected to accelerated thermal stability testing by incubation at 37 °C for 14 days and at 4 °C for 30 days. PCSs at 1000 copies/μL were tested in triplicate, while 1 copy/μL samples were tested in eight replicates. NTCs were tested in 24 replicates. The results demonstrated that NTCs remained valid under both temperature conditions. Ct values and fluorescence peaks of the positive templates at both concentrations showed no significant changes, indicating that the reagents retained stability after thermal stress and were not adversely affected (Table 7 and Figure 3).

#### 2.4.3. Instrument Compatibility

The compatibility of the *Mycoplasma* NAT detection reagents was verified across four different real-time PCR instruments: Thermo Scientific ABI 7500, Thermo Scientific ABI QuantStudio 5, Shanghai Hongshi SLAN-96S, and Roche LightCycler^®^ 480. PCSs at 1000 copies/μL were tested in triplicate, 1 copy/μL samples in eight replicates, and NTCs in 24 replicates. The results confirmed that all NTCs were valid across all instruments. Ct values and fluorescence peaks of the positive samples were consistent across platforms, demonstrating that the reagents are compatible with different qPCR instruments and that test performance is unaffected (Table 8).

### 2.5. Validation of Sample Applicability

#### Application of the NAT Method for *Mycoplasma* Detection

*Mycoplasma* testing is essential during the development and manufacturing of biological products to ensure the absence of microbial contamination. When using the nucleic acid amplification technique (NAT) for *Mycoplasma* detection, rigorous validation is required to confirm that the method is suitable as a supplemental or alternative approach to pharmacopeial methods for *Mycoplasma* release testing. In this study, four biological products (monoclonal antibodies (Table 9), antibody fusion proteins (Table 10), bispecific antibodies (Table 11), and trispecific antibodies (Table 12) were individually spiked with 10 CFU/mL of *Acholeplasma laidlawii* and 10 CFU/mL of *Mycoplasma salivarium* to assess method applicability. Experimental procedures followed the protocols described in Section 4.3. (Sample Nucleic Acid Extraction) and Section 4.4. (qPCR Detection). In parallel, PCS, NCS, and NTC samples were included as quality controls in each experiment. The results showed that all PCS, NCS, and NTCs met acceptance criteria. Furthermore, all spiked biological product samples tested positive for *Mycoplasma*, confirming the method’s applicability for a wide range of biological matrices.

## 3. Discussion

Cell culture is a critical component in the production of many biological products, during which *Mycoplasma* infection can compromise product safety and efficacy [12]. Therefore, *Mycoplasma* detection at every stage of production is essential. According to regulatory guidelines, the cell substrates used in biological product manufacturing must be free of exogenous contaminants, including *Mycoplasma*. Comprehensive monitoring for *Mycoplasma* contamination should be implemented throughout the development process, including raw materials, cell banks, virus seed stocks, unprocessed harvest materials, and final products.

Currently, the Chinese Pharmacopoeia and international regulatory authorities recognize traditional methods for *Mycoplasma* detection, including the culture method and the indicator cell method. However, the culture method is time-consuming, and the indicator cell method has limited sensitivity. These limitations restrict their suitability for rapid release testing of final live-cell products. As a result, NATs have emerged as viable alternatives [13]. When the NAT method achieves a detection sensitivity of 10 CFU/mL, it can serve as a replacement for the culture method; at 100 CFU/mL, it may replace the indicator cell method. The European Pharmacopoeia (EP), Japanese Pharmacopoeia (JP), and United States Pharmacopeia (USP) have all adopted NAT as a recognized *Mycoplasma* detection method. Establishing a suitable *Mycoplasma* detection strategy involves several considerations. If release detection testing is to be conducted on product-related samples, it is necessary to use either pharmacopeia-specified methods or a fully validated NAT method with performance equivalent to traditional methods [14]. Early validation of the detection method is strongly recommended.

However, given the prolonged turnaround time and limited sensitivity of pharmacopoeial methods, it is advisable to implement internal rapid screening techniques, such as PCR or qPCR, to support conditional product release. For the formal adoption of a NAT-based release method, comprehensive validation of the NAT method is essential. In cases where the testing sample material is not intended for product release, such as early-stage research samples or non-final cell banks, rapid methods like loop-mediated isothermal amplification (LAMP) may be used for expedited *Mycoplasma* screening.

To establish a suitable *Mycoplasma* detection method, it is essential to determine whether the test results are intended for reference or for product release, and whether the method complies with regulatory and pharmacopoeial requirements. It is also necessary to evaluate whether method validation has been performed to confirm its sensitivity, specificity, and tolerance [15,16], and whether the method demonstrates comparability to pharmacopoeial procedures.

In addition, known *Mycoplasma* strains should be used as reference substances during detection. By comparing samples against these reference strains, the presence or absence of *Mycoplasma* contamination can be accurately assessed. Standard *Mycoplasma* strains play a vital role in the biopharmaceutical and biological testing industries, and their significance is expected to grow alongside the advancement of biological product development. ChP, EP, JP, and USP all recommend the use of *Mycoplasma hyorhinis*, *Mycoplasma orale*, and *Mycoplasma pneumoniae* as standard strains for LOD validation of NAT methods.

In this study, inactivated *Mycoplasma* strains from the National Collection of Type Cultures (NCTC), UK, were used as standards to assess the sensitivity of the NAT method. The 10 CFU standard *Mycoplasma* dry powder was resuspended in 1 × PBS buffer, followed by nucleic acid extraction and qPCR detection. For each *Mycoplasma* strain, eight replicate extractions and detections were performed per session. The experiment was conducted across three sessions, resulting in a total of 24 detection data points. The LOD was defined as the condition in which at least 23 out of 24 replicates yielded positive PCR results, meeting the acceptance criteria of EP 2.6.7: LOD ≤ 10 CFU/mL. This validated method demonstrated high sensitivity, speed, and convenience for detecting the tested *Mycoplasma* species.

In the methodological validation, this study followed the guidelines outlined in ICH Q2 to validate the *Mycoplasma* NAT method. Specificity testing involved evaluating potential cross-reactivity between the sample matrix and non-*Mycoplasma* bacterial species. Nine commonly used sample matrices in the research and production of biological products were tested, including Dulbecco’s MEM, Ham’s F-10 Medium, M-199 Medium, Ham’s F-12K (Kaighn’s), RMIP-1640 Medium, McCoy’s 5A, Ham’s F-12K (Kaighn’s) with 1.0 mM/L Glutamine, L-15 LEIBOVITZ Medium with 2.05 mM L-Glutamine, DMEM-low glucose (standard type). When spiked with *Spiroplasma citri*, all matrices yielded positive results. All non-spiked control samples were negative, and no cross-contamination was observed during nucleic acid extraction or qPCR detection. These results met the acceptance criteria of EP2.6.7: all spiked samples produced positive detection results, while all non-spiked samples were negative. Regarding cross-reactivity, it is critical to detect a broad range of *Mycoplasma* species to minimize false negatives. Since the detection capability of the NAT method depends on primer and probe design as well as method parameters, potential cross-detection of non-*Mycoplasma* species must also be ruled out. This study therefore included a panel of 14 phylogenetically related non-*Mollicutes* strains, including *Clostridium perfringens*, *Clostridium acetobutylicum*, *Lactobacillus acidophilus*, *Streptococcus mutans*, *Streptococcus pneumoniae*, and other common bacilli, cocci, and fungal organisms such as *Staphylococcus epidermidis*, *Acinetobacter baumannii*, *Enterobacter aeruginosa*, *Micrococcus luteus*, *Pseudomonas aeruginosa*, *Candida albicans*, *Salmonella enterica* subspecies, *Bacillus subtilis*, and *Bacillus cereus*. All tested samples yielded negative results, indicating no cross-reactivity. These results confirm that the complex sample matrix did not interfere with detection and that the method maintained high specificity and reliability in complex systems.

This study also investigated the applicability of the *Mycoplasma* NAT detection method for real biological product samples. The results demonstrated that positive detection was achieved for monoclonal antibodies, antibody fusion proteins, bispecific antibodies, and trispecific antibodies after spiking with *Mycoplasma*-positive standard strains. The validation of sample applicability reflects the method’s detection capability in actual sample matrices, supporting its feasibility for routine testing and enhancing the comprehensiveness of the overall method validation.

This study successfully established a *16S rRNA* primer-based nucleic acid testing (NAT) method and completed preliminary methodological validation, sensitivity assessment, and partial specificity evaluation. Nevertheless, several limitations remain to be addressed in future work.

First, cross-reactivity tests were not further expanded to include phylogenetically closely related non-target species and common laboratory contaminant strains that may interfere with *16S rRNA* primers. Specificity was only evaluated using a limited panel of strains; therefore, potential false-positive risks in complex biological matrices cannot be fully excluded, and the broad-spectrum specificity of the method requires further validation.

Second, methodological validation was performed only under standard laboratory conditions and with routine sample matrices. Systematic evaluation involving more complex practical substrates, different storage conditions, and potential interfering factors was not conducted. The applicability and stability of the assay in large-scale routine testing still need to be verified by enlarging sample sizes and application scenarios.

In addition, this study did not investigate the repeatability and robustness across different experimental batches, operators, and instrument platforms. The anti-interference capability and inter-laboratory compatibility of the method remain to be further evaluated.

In view of the above limitations, future work will supplement cross-reactivity verification of closely related species and contaminant strains, expand the scale of practical sample validation, and improve the robustness evaluation under various experimental conditions. Further optimization of the NAT system will be performed to enhance its practical applicability and reliability.

Experimental data showed that the *Mycoplasma* NAT method satisfied the performance criteria defined in the EP2.6.7-2 NAT validation guidelines. The established method exhibited high sensitivity for detecting *Mycoplasma* contamination and required only a small volume of sample, reducing the overall testing time to approximately 3 h. In addition, this method overcomes limitations associated with traditional pharmacopoeial methods, such as the difficulty in culturing and detecting certain *Mycoplasma* species. As a result, an increasing number of pharmaceutical companies are adopting rapid NAT-based detection methods. NAT methods are also gaining popularity in sterile testing and quality control of residual substances. These trends indicate the growing potential for widespread application of NAT technologies in biopharmaceutical manufacturing.

## 4. Materials and Methods

### 4.1. Materials and Reagents

*Mycoplasma* standard strain:

10CFU™ Sensitivity Standard *Mycoplasma arginini* (Minerva Biolabs (Berlin, German), catalog number: 102-1003).

10CFU™ Sensitivity Standard *Mycoplasma orale* (Minerva Biolabs, catalog number: 102-2003).

10CFU™ Sensitivity Standard *Mycoplasma gallisepticum* (Minerva Biolabs, catalog number: 102-3003).

10CFU™ Sensitivity Standard *Mycoplasma pneumoniae* (Minerva Biolabs, catalog number: 102-4003).

10CFU™ Sensitivity Standard *Mycoplasma synoviae* (Minerva Biolabs, catalog number: 102-5003).

10CFU™ Sensitivity Standard *Mycoplasma fermentans* (Minerva Biolabs, catalog number: 102-6003).

10CFU™ Sensitivity Standard *Mycoplasma hyorhinis* (Minerva Biolabs, catalog number: 102-7003).

10CFU™ Sensitivity Standard *Acholeplasma laidlawii* (Minerva Biolabs, catalog number: 102-8003).

10CFU™ Sensitivity Standard *Spiroplasma citri* (Minerva Biolabs catalog number: 102-9003).

10CFU™ Sensitivity Standard *Mycoplasma salivarium* (Minerva Biolabs, catalog number: 102-1103).

Sample matrices:

Dulbecco’s MEM (Gibco (Thermo Fisher Scientific, Waltham, MA, USA), catalog number: 11965092), Ham’s F-10 Medium (Hyclone (Cytiva, Logan, UT, USA), catalog number: SH30025.01), M-199 Medium (Hyclone, catalog number: SH30253.01), Ham’s F-12K (Kaighn’s) (Gibco, catalog number: 11765054), RPMI-1640 Medium (Gibco, catalog number: 11875093), McCoy’s 5A (Gibco, catalog number: 16600082), Ham’s F-12K (Kaighn’s) + 1.0 mM/L Glutamine (Hyclone, catalog number: SH30026.01), L-15 LEIBOVITZ Medium + 2.05 mM L-Glutamine (Hyclone, catalog number: SH30525.01), DMEM-Low Sugar (Standard type) (Hyclone, catalog number: SH30021.01).

Non-*Mollicutes* strains:

All the strains were procured from Wanjia Standard Materials Co., Ltd. (Xinyang, China). *Staphylococcus epidermidis* (catalog number: BWCC10473), *Acinetobacter baumannii* (catalog number: BWCC10611), *Lactobacillus acidophilus* (catalog number: BWCC10659), *Enteroaerogen* (catalog number: BWCC10548), *Micrococcus luteus* (catalog number: BWCC10820), *Streptococcus mutans* (catalog number: BWCC10533), *Pseudomonas aeruginosa* (catalog number: BWCC10731), *Streptococcus pneumoniae* (catalog number: BWCC10549), *Candida albicans* (catalog number: BWCC10334), *Salmonella enterica subspecies* (catalog number: BWCC11086), *Bacillus subtilis* (catalog number: BWCC10964), *Bacillus cereus* (catalog number: BWCC11116), *Clostridium perfringens* (catalog number: BWCC10343), *Clostridium acetobutylicum* (catalog number: BWCC10192).

Engineering cells/Bacteria and Reagents:

Engineering cells and bacterial DNA:

SF9 DNA (ATCC (Rockefeller, MD, USA), catalog number: CRL-1711); HEK293 DNA, Vero DNA, CHO DNA, *E. coli* DNA, 293T DNA; along with the primers and probes used for *Mycoplasma* detection by the NAT method (fluorescence probe qPCR) were all self-prepared in the laboratory.

Reagents:

Nucleic acid extraction reagent (Yeasen Biotech (Shanghai, China), catalog number: 18700ES70). Monoclonal antibodies, antibody fusion proteins, bispecific antibodies, and trispecific antibodies were provided by Qilu Pharmaceutical Co., Ltd. (Jinan, China).

Primers and probes: 

An alignment of the *Mycoplasma 16S rRNA* sequence was performed to locate regions with specific ends and a conserved central segment. Multiple pairs of forward and reverse primers, as well as corresponding probes, were designed for these regions. Then mix them as the ratio of 1:1:1:1:1:1:1:1:1 (F1:R1:P1:F2:R2:P2:F3:R3:P3, μg/μL).

Forward primer 1 (F1): AAACTTAAAGGAATTGACGGG

Reverse primer 1 (R1): GTTAACCTCCGCTATATCTCTATAGC

Probe 1 (P1): CGCACAAGCGGTGGAGCATGT

Forward primer 2 (F2): TCCTACGGGAGGCAGCAGTA

Reverse primer 2 (R2): CGCGACTGCTGGCACAT

Probe 2 (P2): CATTGTGAAAAATTCC

Forward primer 3 (F3): ACGATGAGAACTAAGTGTTGGCC

Reverse primer 3 (R3): TTCCTTTGAGTTTCATACTTGCGTA

Probe P3 (P3): CTCCGCCTGAGTAGTA

### 4.2. Instruments

Fluorescence quantitative PCR instruments: ABI 7500 (Applied Biosystems (Thermo Fisher Scientific, Carlsbad, CA, USA), ATCC), QuantStudio 5 (Applied Biosystems, ATCC), CFX96 Optic Module (Bio-Rad (Hercules, CA, USA)), SLAN-96S (Shanghai Hongshi Medical Technology Co., Ltd. (Shanghai, China)), LightCycler^®^ 480 (Roche (Basel, Switzerland)).

### 4.3. Nucleic Acid Extraction from Samples

#### 4.3.1. Reconstitution of *Mycoplasma* Strains

According to the instructions provided with Minerva Biolabs’ 10CFU™ Sensitivity Standard, each *Mycoplasma* strain was equilibrated to room temperature. After brief centrifugation, the pellet was resuspended in 1 mL of 1× PBS buffer and incubated at room temperature for 5 min. The suspension was then vortexed for 10 s and centrifuged for 5 s to collect the bacterial cells. A final concentration of 10 CFU/mL was achieved, and the required volume was used for subsequent experiments.

#### 4.3.2. Sample Pretreatment

Ethanol (anhydrous), 1× PBS buffer, and ultrapure water were prepared, and samples were equilibrated to room temperature before processing. The incubation environment was preheated to 60 °C. For initial use, the specified volume of anhydrous ethanol (as indicated on the label) was added to Washing Solutions A* and B*, followed by thorough mixing and proper labeling. Bottles were sealed tightly after each use to prevent ethanol evaporation. Next, 100 μL of the reconstituted *Mycoplasma* suspension (10 CFU/mL) was transferred to a 1.5 mL centrifuge tube. Then, 100 μL of lysis buffer and 10 μL of proteinase K were added. The mixture was vortexed for 10 s and incubated at 60 °C for 20 min. After incubation, 400 μL of binding solution, 9 μL of glycogen, and 6 μL of potassium Poly A salt were added and vortexed. Subsequently, 20 μL of magnetic beads were added, followed by vortexing and incubation for 10 min with intermittent vortexing for 10 s every 3 min. Magnetic beads should be thoroughly vortexed before use to ensure resuspension. After every 4–5 sample additions, the suspension should be remixed to prevent bead aggregation. After brief centrifugation, the tube was placed on a magnetic stand for 1–2 min until the beads were fully captured. The supernatant was carefully discarded. Then, 500 μL of Washing solution A was added, vortexed to disperse beads, centrifuged, and placed on the magnetic stand for 1–2 min before supernatant removal. 500 μL of Washing solution B was added, and the same process was repeated. To remove residual wash buffer, the tube was centrifuged for 10 s, placed on the magnetic stand, and residual liquid was aspirated using a 10 μL pipette. The tube cap was opened and left at room temperature for 3 min to fully evaporate ethanol. Absence of reflection or presence of bead surface cracking indicated complete evaporation. Finally, 50–100 μL of preheated elution buffer (65 °C) was added. The sample was vortexed, centrifuged briefly, and incubated at 65 °C for 5 min with one intermediate mix. After a final centrifugation, the tube was placed on the magnetic stand for 2 min. After the magnetic beads were completely adsorbed, the eluted DNA was transferred to a new tube for downstream PCR or stored at 2–8 °C for up to 1 week. For long-term storage, samples were kept below −18 °C.

### 4.4. Design of Primer-Probe Sets, Species Screening and Sequence Alignment Verification

#### 4.4.1. Design Strategy of Primers and Probes

Sequence specificity analysis and target sequence matching verification of the primers and probes in this study were performed using the online NCBI Primer-BLAST tool (Primer3 (version 2.5.0)). According to the research objectives and practical detection requirements, key design parameters were optimized to ensure sequence matching specificity, while accommodating natural base variations among different bacterial species.

A customized RefSeq whole-genome sequence database containing 183 species from four representative genera of Mollicutes was constructed. Redundant and low-quality sequences were pre-filtered to guarantee the authority, pertinence and accuracy of reference sequences for alignment.

To balance amplification specificity and broad-spectrum coverage capacity, standardized mismatch tolerance criteria were established. The number of mismatches within the last 5 bases at the primer 3′-end was limited to ≤1 to avoid amplification failure caused by mismatches in the critical binding region. The overall mismatch number of the probe was restricted to ≤1, which could adapt to natural base differences among various species and effectively eliminate non-specific binding interference. After multiple rounds of sequence alignment and simulation verification, this parameter system was well optimized to tolerate inherent base variations among the 183 target species and ensure specific binding of each primer-probe set to the target conserved gene sequence.

Three primer-probe sets were designed to target different specific functional regions of conserved genes in Mollicutes. Each set consisted of a forward primer, a reverse primer and a specific fluorescent probe. Primers bound to the upstream and downstream conserved regions of the target gene to initiate amplification, while the probe hybridized to the middle specific region of the gene to realize specific identification and detection of amplified products. A dual recognition strategy of primer anchoring in conserved regions plus probe recognition of specific segments was adopted, and the three sets formed a complementary coverage system. Even if minor base mismatches existed in the binding region of a single set for individual species, effective identification could still be achieved by the remaining primer-probe sets. 

Primer length was controlled within 18–22 bp with a Tm value ranging from 58 °C to 62 °C, which was completely consistent with the actually synthesized primers and subsequent qPCR experimental conditions. Other analytical parameters, including primer dimer prediction, product size range and annealing temperature, adopted the default settings of the tool, strictly simulating routine laboratory qPCR reaction conditions. This ensured high consistency between online alignment predictions and actual amplification performance, improving the reliability and experimental repeatability of primer and probe design.

#### 4.4.2. Definition and Basis of Target Species Screening

The target species range of this study strictly followed the Chinese Pharmacopoeia and relevant industrial guidelines, focusing on clinically and regulatory-concerned microorganisms of the class Mollicutes. It covered four major genera, including *Mycoplasma*, *Ureaplasma*, *Spiroplasma* and *Acholeplasma*, with a total of 183 species. The detection coverage was highly consistent with the specifications of the Pharmacopoeia and industrial regulatory requirements, ensuring standardization and theoretical completeness of the species coverage of the detection system.

#### 4.4.3. Process and Results of Sequence Alignment Verification

Following the establishment of the three primer-probe sets, independent sequence alignment verification was performed one by one on the 183 target species based on the self-built genome database, mismatch tolerance criteria and optimized parameters. Independent alignment was conducted for the forward primer, reverse primer and probe of each set, resulting in a total of 1098 independent alignment analyses. This realized full coverage verification across all target species and primer-probe sets, ensuring systematicness and rigor of the evaluation process.

A unified coverage criterion was defined: each of the 183 target species could be effectively recognized by at least one primer-probe set under the preset mismatch tolerance threshold. The alignment results confirmed that all 183 Mollicutes species could be specifically identified by no less than one primer-probe set without species omission or matching failure. The complete alignment reports, sequence matching screenshots, base mismatch statistics and operation process records have been supplemented as supporting materials, enabling full traceability and rechecking of the verification process.

In summary, this study established a rigorous and comprehensive evaluation system for primer-probe design, involving reference database construction, parameter optimization, mismatch tolerance rule formulation, dual recognition mode design, standardized target species screening and full-coverage sequence alignment verification. The developed system enables stable and broad-spectrum specific detection of 183 Mollicutes species. All original alignment data and verification documents have been fully archived for reference.

### 4.5. qPCR Detection

#### 4.5.1. Preparation of qPCR Reaction System

To prepare the qPCR reaction system, the number of required reaction wells was calculated based on the number of test samples, which included the positive control (PCS), non-template control (NTC), negative sample control (NCS), and the actual test samples (TS). The formula used for determining the total number of reaction wells was: M = (1 × PCS + 1 × NTC + 1 × NCS + N × TS) × 2. This accounted for duplicates of each reaction. The corresponding volume of qPCR Mix was then calculated accordingly, as shown in Table 13.

The qPCR Mix was thoroughly vortexed, followed by low-speed centrifugation to collect any liquid adhering to the cap. Subsequently, 20 μL of the prepared qPCR Mix was dispensed into each well of the reaction plate, and the appropriate sample templates were added to each well in the order shown in Table 14.

#### 4.5.2. qPCR Program Parameter Settings

The qPCR program parameters were set according to standard conditions. The instrument type used was the ABI 7500 configured for a 96 Well plate. The experiment type selected was “Standard Curve” for quantification purposes, using Taqman^®^ reagents as the detection chemistry (Thermo Fisher Scientific, Foster City, CA, USA). The program run time was approximately 2 h under standard cycling conditions. In the “Plate Setup” interface, under the “Define Targets and Samples” section, Target 1, corresponding to the *Mycoplasma 16S rRNA* gene, was assigned to the FAM channel, with FAM as the reporter dye and either MGB or no quencher selected. Target 2, corresponding to the internal control (IC) gene, was assigned to the CY5 channel with CY5 as the reporter dye and no quencher selected. In the “Assign Targets and Samples” section of the “Plate Setup”, “None” was selected for the ROX dye column. The full amplification program parameters are provided in Table 15.

## 5. Conclusions

This study successfully developed and thoroughly validated a *Mycoplasma* NAT detection method based on multiplex fluorescence quantitative PCR. The method demonstrated a limit of detection (LOD) of 10 CFU/mL, exhibited strong specificity, and showed no cross-reactivity with various matrices or non-*Mycoplasma* microorganisms. The reagents displayed excellent stability and tolerance, with consistent performance across multiple qPCR platforms. Furthermore, the method demonstrated effective detection in spiked real-world biological product samples. The validation results confirmed that this NAT-based method complies with relevant technical guidelines and offers a fast, sensitive, and reliable alternative for process monitoring and lot-release detection of biological products.

## Figures and Tables

**Table 1:** Comparison Table of Key Parameters for *Mycoplasma* NAT Detection Methods.

Detection Method	Amplicon Length (bp)	Number of Primer/Probe Sets	Number of Covered Species (Mollicutes)	Limit of Detection (LOD)	Matrix Applicability Range	Literature/Source Citation
This Study (Three Primer-Probe System)	100–200 bp	3 sets	183 species	Single copy	Pharmaceutical production-related matrices (e.g., cell cultures, bulk biopharmaceuticals), pharmacopoeial reference strains	This Study
Early *16S rRNA*-Targeted Method (Degenerate Primers)	Long (usually >300 bp)	1–2 sets (degenerate primers)	Broad coverage of Mollicutes (specific number not specified)	Low (mostly 10^2^–10^3^ copies)	Basic microbial samples (e.g., cultured bacterial broth)	[8]
Pharmacopoeial Standard Species-Specific Method	Short (usually 80–150 bp)	1 set per target species	Sigle or a few specific species (e.g., *Mycoplasma pneumoniae*, *Mycoplasma fermentans*)	High (1–10 copies)	Pharmaceutical matrices, clinical samples (partially applicable)	[10]
Multiplex Detection Method (Triple or More Sets)	Medium (100–200 bp)	≥3 sets	Relatively broad coverage (usually 50–120 species)	Medium (10–10^2^ copies)	Multiple types of microbial samples, pharmaceutical matrices	[11]
Bioinformatics-Optimized Primer Method	100–180 bp	1–2 sets	Moderately broad coverage (30–80 species)	Approx. 10 copies	Laboratory-purified samples, partial pharmaceutical matrices	[9]

Notes: 1. The “amplicon length” of this study can be supplemented with actual experimental data, and the range in the table is the recommended optimal length for fluorescent PCR; 2. The LOD of each method is based on typical values reported in the literature, and specific values may vary slightly depending on the detection instrument and reagent batch; 3. The matrix applicability range is summarized based on the method design objectives and verification scenarios, and can be further refined according to research needs.

**Table 2:** Current Requirements for *Mycoplasma* Detection and Method Verification in Chinese and Foreign Regulations.

S/N	Institution/Standard	European Pharmacopoeia <2.6.7>	Japanese Pharmacopoeia <G3-14-170>	United States Pharmacopoeia <63>	Chinese Pharmacopoeia 2020 <3301>	WHO
	Validation Parameters	Specificity, LOD, Tolerance	Specificity, LOD, Tolerance	Sensitivity	Undefined	(Semi) Quantitative and Qualitative Experiments for Detecting Sensitivity
1	*Mycoplasma* strains to be verified	*Acholeplasma laidlawii*	*Acholeplasma laidlawii*	*Acholeplasma laidlawii*	\	*Acholeplasma laidlawii*
2		*Mycoplasma fermentans*	*Mycoplasma fermentans*	*Mycoplasma fermentans*	\	*Mycoplasma fermentans*
3		*Mycoplasma hyorhinis*	*Mycoplasma hyorhinis*	*Mycoplasma hyorhinis*	*Mycoplasma hyorhinis* ***	\
4		*Mycoplasma orale*	*Mycoplasma orale*	*Mycoplasma orale*	*Mycoplasma orale*	*Mycoplasma orale*
5		*Mycoplasma pneumoniae*	*Mycoplasma pneumoniae*	*Mycoplasma pneumoniae*	*Mycoplasma pneumoniae*	*Mycoplasma pneumoniae*
6		*Mycoplasma gallisepticum*	\	*Mycoplasma gallisepticum*	\	\
7		*Mycoplasma synoviae* *	*Mycoplasma synoviae*	*Mycoplasma synoviae*	\	\
8		*Mycoplasma arginini*	*Mycoplasma arginini*	\	\	\
9		*Spiroplasma* **	*Spiroplasma*	\	\	\
10		\	*Mycoplasma salivarium*	\	\	\

* If avian cells or materials are used or come into contact during the production process, LOD validation for *Mycoplasma synoviae* should be conducted. ** If insect or plant materials are used or come into contact during the production process, LOD validation for *Spiroplasma* should be conducted. *** If the proportion of *Mycoplasma hyorhinis* in contaminated samples is unusually high, it may draw the attention of the China National Institutes for Food and Drug Control.

**Table 3:** LOD Results.

No.	Name of *Mycoplasma*	Concentration of Bacterium	Detection Status				Detection Rate
			Experiment 1	Experiment 2	Experiment 3	Total	
1	*Mycoplasma arginini*	10 CFU/mL	8/8	8/8	8/8	24/24	100%
2	*Mycoplasma orale*	10 CFU/mL	8/8	8/8	8/8	24/24	100%
3	*Mycoplasma gallisepticum*	10 CFU/mL	8/8	8/8	8/8	24/24	100%
4	*Mycoplasma pneumoniae*	10 CFU/mL	8/8	8/8	8/8	24/24	100%
5	*Mycoplasma synoviae*	10 CFU/mL	8/8	8/8	8/8	24/24	100%
6	*Mycoplasma fermentans*	10 CFU/mL	8/8	8/8	8/8	24/24	100%
7	*Mycoplasma hyorhinis*	10 CFU/mL	8/8	8/8	8/8	24/24	100%
8	*Acholeplasma laidlawii*	10 CFU/mL	8/8	8/8	8/8	24/24	100%
9	*Spiroplasma citri*	10 CFU/mL	8/8	8/8	8/8	24/24	100%
10	*Mycoplasma salivarium*	10 CFU/mL	8/8	8/8	8/8	24/24	100%

**Table 4:** Results of sample matrix interference.

Sample Type		Mean Ct of Target (FAM) Channel	Mean Ct of Internal Reference (CY5) Channel	Result Judgment
9 common sample matrices + DNA diluent	Dulbecco’s MEM	NA, No obvious peak	26.84	Negative
	Ham’s F-10 culture medium	NA, No obvious peak	26.78	Negative
	M-199 Medium	NA, No obvious peak	26.76	Negative
	Ham’s F-12K (Kaighn’s)	NA, No obvious peak	26.86	Negative
	RPMI-1640 Medium	NA, No obvious peak	26.74	Negative
	McCoy’s 5A	NA, No obvious peak	26.78	Negative
	Ham’s F-12K (Kaighn’s) + 1.0 mM/L Glutamine	NA, No obvious peak	26.76	Negative
	L-15 LEIBOVITZ MEDTA + 2.05 mM L-Glutamine	NA, No obvious peak	26.73	Negative
	DMEM-low glucose (standard type)	NA, No obvious peak	26.81	Negative
	DNA diluent	NA, No obvious peak	26.88	Negative
(9 common sample matrices + DNA diluent) spiked	Dulbecco’s MEM + 10 CFU/mL *Spiroplasma citri*	30.23	26.41	Positive
	Ham’s F-10 culture medium + 10 CFU/mL *Spiroplasma citri*	30.27	26.45	Positive
	M-199 Medium + 10 CFU/mL *Spiroplasma citri*	30.20	26.49	Positive
	Ham’s F-12K (Kaighn’s) + 10 CFU/mL *Spiroplasma citri*	30.41	26.57	Positive
	RPMI-1640 Medium + 10 CFU/mL *Spiroplasma citri*	30.41	26.50	Positive
	McCoy’s 5A + 10 CFU/mL *Spiroplasma citri*	30.34	26.62	Positive
	Ham’s F-12K (Kaighn’s) + 1.0 mM/L Glutamine + 10 CFU/mL *Spiroplasma citri*	30.33	26.60	Positive
	L-15 LEIBOVITZ MEDTA + 2.05 mM L-Glutamine + 10 CFU/mL *Spiroplasma citri*	30.48	26.57	Positive
	DMEM-low glucose (standard type) + 10 CFU/mL *Spiroplasma citri*	30.59	26.58	Positive
	DNA diluent + 10 CFU/mL *Spiroplasma citri*	30.60	26.69	Positive
Positive control	PCS	24.68	26.38	Positive
Negative control	NCS	NA, No obvious peak	26.66	Negative
	NTC	NA, No obvious peak	26.59	Negative

**Table 5:** Detection results of cross-reactivity.

Sample Type		Mean Ct of Target (FAM) Channel	Mean Ct of Internal Reference (CY5) Channel	Result Judgment
Fungi	*Staphylococcus epidermidis* 10 ng/test	NA, No obvious peak	26.63	Negative
	*Clostridium perfringens* 0.1 ng/test	Ct > 40	27.06	Negative
	*Clostridium acetobutylicum* 0.1 ng/test	NA, No obvious peak	27.08	Negative
	*Baumannii* 10 ng/test	NA, No obvious peak	26.56	Negative
	*Lactobacillus acidophilus* 10 ng/test	NA, No obvious peak	26.77	Negative
	*Enteroaerogen* 10 ng/test	NA, No obvious peak	26.79	Negative
	*Micrococcus luteus* 10 ng/test	NA, No obvious peak	26.37	Negative
	*Streptococcus mutans* 10 ng/test	NA, No obvious peak	26.55	Negative
	*Pseudomonas aeruginosa* 10 ng/test	NA, No obvious peak	27.07	Negative
	*Streptococcus pneumoniae* 10 ng/test	NA, No obvious peak	26.82	Negative
	*Candida albicans* 10 ng/test	NA, No obvious peak	26.78	Negative
	*Salmonella enterica* subspecies enteritis 10 ng/test	NA, No obvious peak	26.73	Negative
	*Bacillus subtilis* 10 ng/test	NA, No obvious peak	26.52	Negative
	*Bacillus cereus* 10 ng/test	NA, No obvious peak	26.68	Negative
Engineering cell	HEK293 DNA 30 ng/test	NA, No obvious peak	26.91	Negative
	Vero DNA 30 ng/test	NA, No obvious peak	26.83	Negative
	CHO DNA 30 ng/test	NA, No obvious peak	26.90	Negative
	*E. coli* DNA 30 ng/test	NA, No obvious peak	26.73	Negative
	293T DNA 30 ng/test	NA, No obvious peak	26.90	Negative
	Sf9 DNA 30 ng/test	NA, No obvious peak	26.83	Negative
Positive control	PCS	24.82	26.66	Positive
Negative control	NCS	NA, No obvious peak	26.81	Negative
	NTC	NA, No obvious peak	26.85	Negative

**Table 6:** Detection results of freeze–thaw stability.

Freeze–Thaw Cycles	0 Cycle		20 Cycles	
Sample type	Mean Ct of target (FAM) channel	Mean Ct of internal reference (CY5) channel	Mean Ct of target (FAM) channel	Mean Ct of internal reference (CY5) channel
1000 copies/μL PCS	24.51	26.45	24.49	26.55
1 copies/μL PCS	33.79	26.73	34.24	26.93
NTC	NA, No obvious peak	26.67	NA, No obvious peak	26.86

**Table 7:** Detection results of thermal acceleration stability.

Thermal Acceleration Treatment	Control		37 °C for 14 Days		4 °C for 30 Days	
Sample type	Mean Ct of target (FAM) channel	Mean Ct of internal reference (CY5) channel	Mean Ct of target (FAM) channel	Mean Ct of internal reference (CY5) channel	Mean Ct of target (FAM) channel	Mean Ct of internal reference (CY5) channel
1000 copies/μL PCS	23.98	26.41	24.30	26.47	24.28	26.36
1 copies/μL PCS	33.74	26.54	33.97	26.90	33.64	26.56
NTC	NA, No obvious peak	26.54	NA, No obvious peak	26.56	NA, No obvious peak	26.76

**Table 8:** Detection results of instrument applicability.

Instrument Model	Thermo Scientific, ABI 7500		Thermo Scientific, Q5		Hongshi, SLAN-96S		Roche, LightCycler^®^ 480	
Sample type	Mean Ct of target (FAM) channel	Mean Ct of internal reference (CY5) channel	Mean Ct of target (FAM) channel	Mean Ct of internal reference (CY5) channel	Mean Ct of target (FAM) channel	Mean Ct of internal reference (CY5) channel	Mean Ct of target (FAM) channel	Mean Ct of internal reference (CY5) channel
1000 copies/μL PCS	24.60	26.68	26.17	25.57	26.89	21.63	26.83	26.34
1 copies/μL PCS	34.20	26.82	35.66	25.62	36.55	21.73	36.26	26.37
NTC	NA, No obvious peak	26.94	NA, No obvious peak	25.62	NA, No obvious peak	21.80	NA, No obvious peak	26.40

**Table 9:** Applicability detection results of monoclonal antibody samples.

Detection Value of Samples	Ct Value of the Signal Pathway		Results
	FAM	CY5	
monoclonal antibody + + *Acholeplasma laidlawii* 10 CFU/mL	31.771	26.091	Positive
	31.515	25.95	
monoclonal antibody + + *Mycoplasma salivarium* 10 CFU/mL	31.245	25.979	Positive
	30.776	26.112	
PCS	23.829	25.641	Positive
	23.782	25.595	
NCS	/	25.842	Negative
	/	25.926	
NTC	/	26.073	Negative
	/	25.94	
FAM		IC	
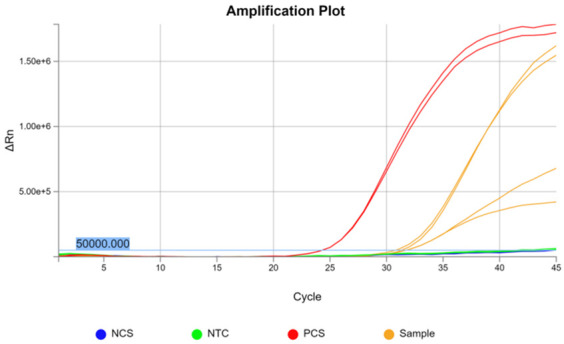		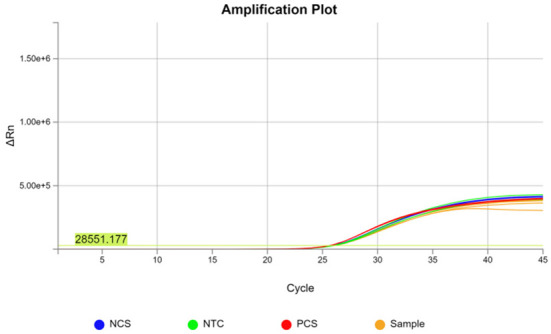	

**Table 10:** Applicability detection results of fusion protein samples.

Detection Value of Samples	Ct Value of the Signal Pathway		Results
	FAM	CY5	
Fusion protein + *Acholeplasma laidlawii* 10 CFU/mL	31.584	26.01	Positive
	31.979	26.044	
Fusion protein + *Mycoplasma salivarium* 10 CFU/mL	30.759	26.131	Positive
	30.849	25.987	
PCS	23.829	25.641	Positive
	23.782	25.595	
NCS	/	25.842	Negative
	/	25.926	
NTC	/	26.073	Negative
	/	25.94	
FAM		IC	
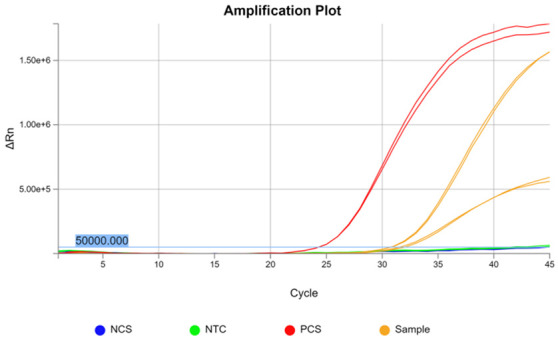		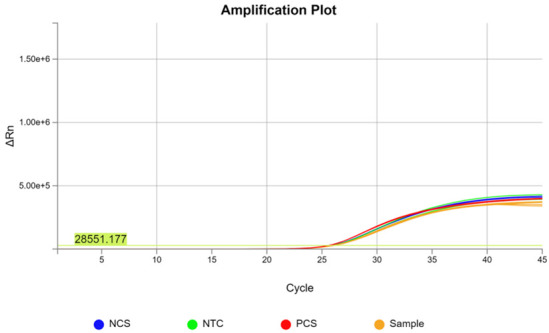	

**Table 11:** The applicability detection results of the bispecific antibody samples.

Detection Value of Samples	Ct Value of the Signal Pathway		Results
	FAM	CY5	
bispecific antibody + *Acholeplasma laidlawii* 10 CFU/mL	32.037	26.053	Positive
	31.61	26.171	
bispecific antibody + *Mycoplasma salivarium* 10 CFU/mL	30.727	26.033	Positive
	30.885	26.177	
PCS	23.829	25.641	Positive
	23.782	25.595	
NCS	/	25.842	Negative
	/	25.926	
NTC	/	26.073	Negative
	/	25.94	
FAM		IC	
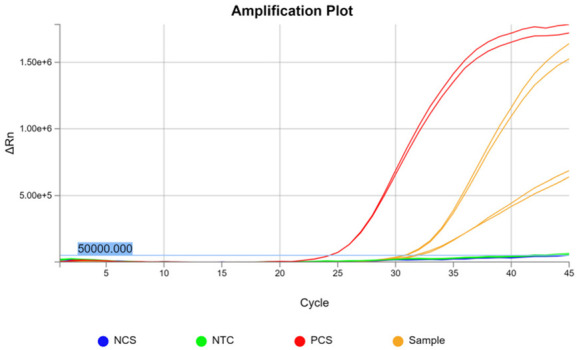		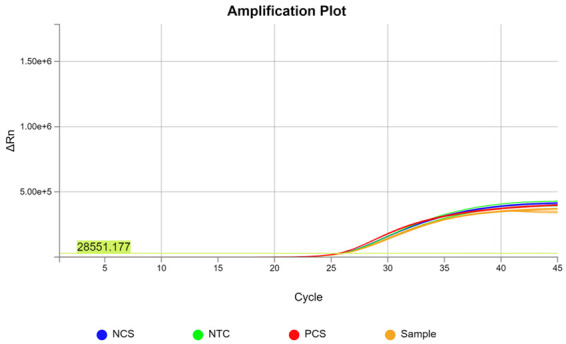	

**Table 12:** Applicability detection results of trispecific antibody samples.

	Ct Value of the Signal Pathway		Results
	FAM	CY5	
trispecific antibody + *Acholeplasma laidlawii* 10 CFU/mL	31.832	26.115	Positive
	31.829	26.064	
trispecific antibody + *Mycoplasma salivarium* 10 CFU/mL	30.948	26.268	Positive
	30.746	26.064	
PCS	23.829	25.641	Positive
	23.782	25.595	
NCS	/	25.842	Negative
	/	25.926	
NTC	/	26.073	Negative
	/	25.94	
FAM		IC	
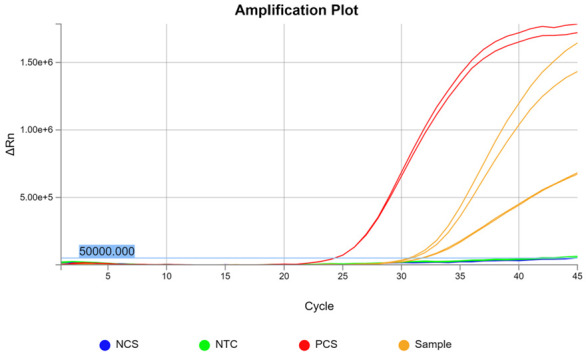		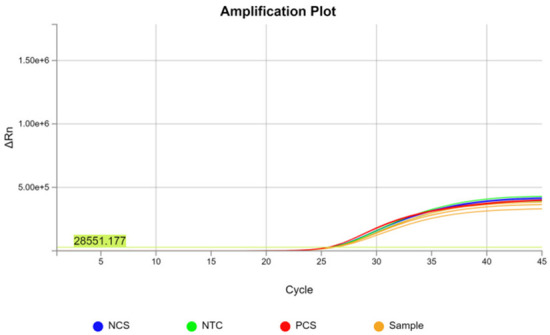	

**Table 13:** Preparation of the qPCR reaction system.

Component	Volume (Single Well)	Volume (M Well)
2× MyqPCR Reaction Buffer	15 μL	(M + 2) × 15 μL
MyPrimer & Probe MIX	4 μL	(M + 2) × 4 μL
Internal control (IC)	1 μL	(M + 2) × 1 μL
Total	20 μL	(M + 2) × 20 μL

**Table 14:** Sample loading example.

Sample	Total Reaction Solution in Each Tube or Well is 30 μL
TS	20 μL qPCR Mix + 10 μL purified solution of the test sample
NTC	20 μL qPCR Mix +10 μL DNA diluent
NCS	20 μL qPCR Mix + 10 μL NCS purified solution
PCS	20 μL qPCR Mix + 10 μL positive control

**Table 15:** Standard amplification program settings.

No.	Reaction Stage	Temperature	Time	Cycles
1	Predegeneration	95 °C	5 min	1
2	Degeneration	95 °C	15 s	45
3	Annealing/extension (fluorescence signal collection)	62 °C	30 s	

## Data Availability

The original contributions presented in this study are included in the article. Further inquiries can be directed to the corresponding author.
